# Orally Dispersible Dosage Forms for Paediatric Use: Current Knowledge and Development of Nanostructure-Based Formulations

**DOI:** 10.3390/pharmaceutics14081621

**Published:** 2022-08-03

**Authors:** Andreea Cornilă, Sonia Iurian, Ioan Tomuță, Alina Porfire

**Affiliations:** Department of Pharmaceutical Technology and Biopharmacy, Faculty of Pharmacy, “Iuliu Haţieganu” University of Medicine and Pharmacy, 41 V. Babes Street, 400012 Cluj-Napoca, Romania; ioana.an.cornila@elearn.umfcluj.ro (A.C.); tomutaioan@umfcluj.ro (I.T.); aporfire@umfcluj.ro (A.P.)

**Keywords:** orodispersible dosage forms, nanostructure, polymeric nanoparticles, taste-masking, oral bioavailability, paediatric medicines

## Abstract

The paediatric population has always suffered from a lack of medicines tailored to their needs, especially in terms of accurate dosage, stability and acceptability. Orodispersible dosage forms have gone through a resurrection as an alternative to liquid formulations or fractioned solid formulations, although they are still subject to several inconveniences, among which the unpleasant taste and the low oral bioavailability of the API are the most significant hurdles in the way of achieving an optimal drug product. Nanostructures can address these inconveniences through their size and variety, owing to the plethora of materials that can be used in their manufacturing. Through the formation and functionalisation of nanostructures, followed by their inclusion in orodispersible dosage forms, safe, stable and acceptable medicines intended for paediatric use can be developed.

## 1. Introduction

One of the most frequent challenges encountered in the field of paediatric pharmacotherapy is the need for easily adjustable dosage forms, which has been addressed for a long time through the development and usage of liquid preparations in the form of oral solutions (including syrup-based), suspensions and emulsions. However, nothing in this field is one-size-fits-all; hence, this type of formulation is not suitable for every active pharmaceutical ingredient, mainly because of the impending stability issues that occur within liquid media, such as degradation and microbial contamination. To avoid this, pharmacists around the world have resorted to adaptations of solid dosage forms they found suitable, crushing tablets and administering the resulting powders either as capsules or on their own, mixed with a sweetening agent (e.g., glucose) or into soft foods or beverages; this is an approach that is still subject to physico-chemical incompatibilities and acceptability issues.

The year 2007 was marked by the implementation of the European Paediatric Regulation (EC No. 1901/2006), which incentivises pharmaceutical companies to involve paediatric populations in their studies during the development of medicinal products with new active pharmaceutical ingredients (APIs), under a new dosage form or with a new indication [1,2]. Similarly, the Food and Drug Administration of the United States of America (FDA) has issued the Best Pharmaceuticals for Children Act (BPCA) and the Pediatric Research Equity Act (PREA), which demand an ethical inclusion of children in drug development and clinical trials [3].

Since these regulations came into effect, the field of paediatric drug development has slowly but steadily taken off, with one of the most important advancements in this field being the rediscovery of orodispersible tablets and granules as patient-friendly dosage forms, along with the optimisation of processes used in their manufacturing and that of other forms, namely the oral lyophilisates and the orodispersible films. While all of these forms are more compatible with the administration to younger populations than other solid dosage forms and extend the stability of APIs compared to liquid formulations, they still encounter some shortcomings, most of which could be solved through the use of nanosystems. This review article aims to be the first to present the way nanostructures and orodispersible dosage forms may come together into modern oral dosage forms intended for paediatric use. It addresses the latest research developments, as well as the current situation regarding the authorised medicines in this category. Moreover, it encourages further research in this field through the introduction of a quality profile designed specifically for orodispersible medicines for paediatric use and highlights several under-examined subjects regarding the manufacturing of oral dosage forms that include nanostructures.

## 2. Orally Dispersible Dosage Forms (ODx)—General Aspects

**Figure 1:**
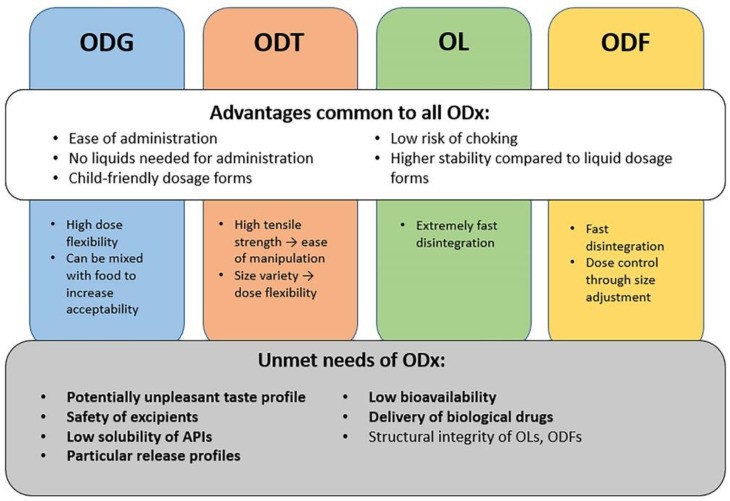
Advantages of ODx and unmet needs of these formulations. ODG—orodispersible granules; ODT—orodispersible tablet; OL—oral lyophilisate; ODF—orodispersible film.

Figure 1 describes the common and specific advantages of every orally disintegrating dosage form, along with the challenges encountered in current formulations that need to be addressed to improve their stability, safety and efficacy, which are particularly important in paediatric pharmacotherapy.

### 2.1. Granules (ODGs)

Granules are multiparticulate dosage forms that are usually further processed into capsules or tablets but can also be used as such. From a paediatric-friendly formulation viewpoint, their reduced size is their main asset, allowing for a minimum-risk swallowing process, either on their own or mixed with soft foods and beverages, regardless of the patient’s age. The multiparticulate aspect of this form enables the possibility of finely adjusting the doses, which is a crucial aspect in paediatric pharmacotherapy, especially in younger children [4,5]. Any currently used granulation process can be employed in the manufacturing of ODGs, as long as the excipients and method are suitable for the desired formulation and compatible with the API [4,5,6].

In the European Pharmacopoeia, ODGs are not a standalone dosage form, following the specifications in the Granules monograph [5,7]. Hence, the quality control process may not be very well adapted to this particular dosage form: a common issue for most orodispersible dosage forms. As an example, the Granules monograph mentions a disintegration time test solely for effervescent granules, which are supposed to disintegrate within 5 min. Other types of granules are supposed to be swallowed as such, for example, tablets or capsules, according to this monograph, although they lack a disintegration time assessment [5,7].

Since granules are a solid dosage form, their stability is superior to that of liquid formulations, but they are still subject to degradation due to atmospheric humidity. Therefore, oral and orodispersible granules are frequently packaged in single-dose sachets whose contents can only be used immediately upon opening [4,6].

Another significant disadvantage of ODGs is the displeasing taste profile induced by the API, which can lead to low acceptability from patients, especially paediatric. To improve this aspect, processes such as microencapsulation and coating can be employed [5].

### 2.2. Tablets (ODTs)

The European Pharmacopoeia describes orodispersible tablets as uncoated tablets that rapidly disintegrate in the oral cavity before being swallowed [8], turning into a solution or a suspension using the existing saliva and rarely requiring the use of external liquids such as water. For the orodispersible aspect to not become a hassle, the European Pharmacopoeia requires a disintegration time of under three minutes in water as a prerequisite for ODTs [8], while the FDA defines ODTs as forms that disintegrate in under thirty seconds in an in vitro setting [9]. ODTs are regarded as superior dosage forms in terms of acceptability, safety of administration and sometimes expected drug bioavailability, or at least pharmacokinetic similarity [3,10,11].

There are several methods used in the preparation of ODTs, such as compression, sublimation, phase transition, extrusion, moulding, etc. [5,12].

Compression is largely preferred as a manufacturing process for ODTs because of its multiple advantages: the process is well understood, cost-efficient, easy to transfer from laboratory to industrial scale and the resulting tablets are sturdy, able to handle a high drug load and easily reproducible [13]. However, the porous structure needed for quick disintegration is difficult to achieve through compression [12] while simultaneously maintaining tablet integrity, especially during transportation and handling.

Direct compression is preferred instead of granulation due to its lower costs, but more difficult to implement in the production of ODTs, because it needs higher compaction pressures for the powder particles to aggregate properly into a physically resistant tablet. This negatively impacts the disintegration of the final product due to the smaller gaps between the compressed particles that make the tablet harder to penetrate by saliva [12].

The multifunctionality of co-processed excipients can come as a substantial advantage in the manufacturing of ODTs, on account of the multiple categories of disintegration promoters they can contain, ranging from highly water-soluble diluents to superdisintegrants. Among the most frequently used excipients for this method there are commercial names such as Ludipress^®^, Ludiflash^®^ or Ran-Explo-S^®^ [5,12,14].

Several co-processed excipients designed for the manufacturing of ODTs have been studied both in terms of their manufacturability and patient acceptability [15,16]. The manufacturability tests included the determination of the Carr index, compaction analysis and tensile strength, friability, disintegration and fineness of dispersion tests.

Mannitol-based co-processed excipients performed well in studies investigating their compressibility, mechanical resistance and disintegration profile, as well as regarding their taste-masking capabilities [15,16,17,18].

Other patented formulation approaches described in the literature were Orasolv^®^ or Durasolv^®^, based on effervescence [19]. One of the key aspects of these two formulations was the use of materials with a very reduced particle size, which grants a quick disintegration within the oral cavity without the nuisance of an unpleasant mouthfeel afterwards [12,20].

Due to their high-water solubility and taste-improving capabilities, saccharides and sugar alcohols have always been of interest in the field of orodispersible formulations. Hence, several excipient combinations created for the preparation of ODx include mono- or polysaccharides and/or their respective alcohols. Sugars and sugar alcohols play an important part in the formulations of ODTs, while also being used in other less commonly used processes for ODT manufacturing such as the phase transition and candy floss methods, both based on the melting of these compounds [12,20].

There are a few compression-adjacent techniques that can be employed to obtain ODTs, such as the sublimation and solid dispersion methods. The sublimation method involves the inclusion of volatile components, for example, camphor, or urea, in the compression mixture, which after compression would be removed under a vacuum to leave behind a porous structure that will be easily infiltrated by the saliva [20].

The moulding or solid dispersion method has multiple approaches, some of which are used in the manufacturing of ODTs. An application of this process was the development of multi-channel ODTs, which involves the use of a custom-made compression to obtain a tablet that exhibits multiple macroscopic channels that would enhance the penetration of saliva [21,22].

Even though a plethora of tablet manufacturing methods have been developed over the years, compression has remained the most largely used, due to its efficiency, robustness and scale-up capabilities [17,23,24,25,26,27]. Hence, the current research in the field of tablet manufacturing intends to optimise the compression process and exploit the potential of already existing excipients rather than developing entirely new methods.

An important tool in the field of tablet pre-formulation, including the ODTs, is the use of the SeDeM expert system: an algorithm meant to help in the choice of compression excipients to diminish the impact of the poor compressibility of an API. This system is based on a set of measurements that should be assessed for the excipients and drug powders to determine the interactions that may occur. Among the benefits, it can accelerate pre-formulation stages in the ODT development and avoids the waste of excipients and APIs [28,29].

Nevertheless, newer technologies are currently being customised for the manufacturing of ODTs. Among those, 3D printing is the most popular, having already produced a commercially available dosage form in the United States of America, Spritam^®^, marketed as a fast-melt tablet that only needs a very small amount of water to disintegrate in the oral cavity [30]. Given this success and the high customisability of the products obtained as a result of three-dimensional printing [31], this technology gained a lot of interest as an oral dosage form manufacturing method, including in the field of ODTs [32]. In the attempt to obtain highly porous structures with large surface areas and quick disintegration, electrospun microfibres were produced and subsequently compressed to ODTs with benefits for the BCS Class II API solubility [33,34]. At the same time, new excipients are emerging as well, such as polysaccharides of vegetal origin used as superdisintegrants [35].

Along with the risk of low mechanical strength imposed by low-force compression processes, ODTs are subject to hygroscopicity, usually imparted by the excipients in the formulation, that impose the use of special packaging [3,5]. However, a larger number of excipients, that are not uncommon for tablet formulations, can become a safety concern in the case of paediatric medicines, since paediatric patients can suffer from adverse effects because of certain excipients [5].

### 2.3. Minitablets (ODMTs)

Minitablets are a subcategory of tablets defined by their reduced size (under 5 mm in diameter), that can easily and safely be swallowed and have been found to present high acceptability in clinical studies, even in neonates [36]. Minitablets benefit from their size, in the same way granulates do: the possibility to administer multiple units at a time allows for a highly precise and flexible-dose control that does not impose any manipulation of dosage forms. This is advantageous in the event of administering medication to paediatric patients, especially those of younger ages [37]. The orodispersible tablet preparation methods have been scaled to produce orodispersible minitablets, using the same excipients and technologies, therefore ensuring a greater safety of administration and acceptability [38]. The reduced dimensions of the ODMTs make compression the sole suitable process for their manufacturing, although it is not fully compatible with the dosage form and must be adapted, e.g., adjusting the compaction pressure to lower values than in the case of regularly sized tablets in order not to hinder the disintegration process.

The effects of compaction pressure and the use of certain co-processed excipients on the quality profile of ODMTs have been studied several times. Lura et al. conducted studies on the capacity of Ludiflash and galenIQ™ (isomalt-based co-processed excipient) to be used for the manufacturing of ODMTs [39], later optimising the process for scale-up from laboratory to industrial scale [40]. From the same research group, Kokott et al. proved the capacity of Parteck ODT and Hisorad to be compressed into sturdy ODMTs that disintegrate quickly, both in a compaction simulator and on an industrial-grade tablet press [41].

Due to their very compact nature, ODMTs might need different disintegrants than those commonly used in tablets. Soulairol et al. prepared xerogels from alginic acid and calcium alginate to take advantage of their swelling capacities for the faster disintegration of ODMTs containing mannitol, and microcrystalline cellulose and magnesium stearate. Even though these novel excipients need to be further developed, they proved to be promising in terms of both compression behaviour and disintegration enhancement [42]

Since 3D printing has been discussed as a tablet manufacturing process, it is worth mentioning that extrusion-based 3D printing and moulding have been studied as ODMT production methods as well [43,44].

ODMTs are subject to the same drawbacks as granules, such as dosing errors or the contamination or untimely disintegration of tablets that could occur upon hand counting. To avoid this, minitablets can be packed in sachets according to the required dose, or certain apparati, such as the Smart Mini Tablet Dispenser (sMTS), can be employed as a way to quickly and accurately count the minitablets needed in a dose [45]. A similar device is Sympfiny, a syringe-like apparatus designed for the quick administration of multiparticulates that can be attached to certain containers [46].

### 2.4. Oral Lyophilisates (OLs)

The oral lyophilisate is considered by some to be a particular type of ODT, due to its similar shape and packaging, although the manufacturing process is unrelated to any form of compression. The European Pharmacopoeia defines the OL as a solid dosage form obtained through the freeze-drying (lyophilisation) of a liquid or semi-solid preparation, intended to disintegrate rapidly in the mouth to form a solution or a suspension [8]. Usually, the freeze-drying process takes place directly in the blister used as primary packaging for the final dosage form [5].

Freeze-drying or lyophilisation, a process commonly used for the drying of thermolabile materials or as a way to ensure a longer shelf life for certain drug products [47], can also be employed in the manufacturing of oral dosage forms in the shape of tablet-like porous matrixes, with a very short disintegration time [47].

Moreover, if the API is soluble in the aqueous dispersion, lyophilisation could render it in an amorphous state, enabling faster dissolution compared to the crystalline form [12] and thus, higher bioavailability [5].

The most frequently used excipients in the formulation of oral lyophilisates are matrix forming excipients, such as gelatin, alginates or amino acids, of which glycine is the most common; these maintain the structural integrity of the matrix after the removal of the liquid components [48]. Several other excipients have been tested as alternatives for gelatin in OL formulations: PVP [49], xanthan gum [50] and PVA [51]. Fenugreek seed mucilage in several concentrations has been tested as a matrix-forming agent in an OL formulation, with good results at lower concentrations [52].

Surfactants ensure proper wetting upon administration, while thickening agents improve the uniformity of the final dosage form. Bulking agents, intended to add heft to the lyophilisate, may also bring the further advantages of cryoprotection and taste-masking, as is the case of mannitol, one of the most frequently used excipients for this application [47,53]. Maltodextrin has been proven to be a suitable bulking agent as well, in the case of an ibuprofen OL formulation, leading to a sturdy product that could easily be manipulated, while keeping a sufficiently short disintegration time and a good dissolution profile [54]. Not only the excipients, but also the freeze-drying parameters, impact the final product. The freezing rate and the inclusion of an annealing protocol can influence pore size and the API crystalline state with consequences on disintegration, mechanical strength and API dissolution [55]. Several formulation strategies for OLs have been patented, such as Zydis^®^, which involves the inclusion of the API into a gelatin dispersion, the addition of mannitol or ion-exchange resins to avoid both structural collapse and the taste issues and other excipients like flavouring agents or preservatives. The resulting lyophilisate must be sealed immediately after the freeze-drying process ends, because of the poor stability of the resulting tablets in both high temperatures and humid conditions [48]. Another reported technology is Lyoc^®^, which uses a large quantity of sugars or sugar alcohols, along with other excipients, to guarantee the homogeneity and structural integrity of the tablets. The process is similar to other lyophilisation-based technologies, and specifically addressed to low-solubility drugs, which are turned into nanoparticles prior to being mixed with the other excipients. Hence, the resulting product disintegrates rapidly into a nanosuspension with particles up to 500 nm [56].

The main disadvantage of the OL is the low mechanical strength that makes the manipulation prior to administration difficult [5]. Moreover, due to the nature of the excipients and the thorough drying process, the final product is highly hygroscopic. On account of the technology used and the packaging needed to maintain the structural integrity and stability of the dosage form [57], the manufacturing of OLs imposes higher costs compared to compressed ODTs [5].

### 2.5. Films (ODFs)

Orodispersible films are defined in the European Pharmacopoeia as solid oromucosal preparations consisting of one or multiple layers that disperse and release the active substance rapidly [58]. ODFs have been developed as a solution to the shortcomings encountered in other oral dosage forms: the low mechanical strength of ODTs, the reduced swallowability of regular tablets and capsules in paediatric and/or dysphagic patients or the lack of dose uniformity that might be seen in liquid formulations, especially suspensions [59]. Furthermore, this dosage form benefits from increased dose flexibility, the patient or caregiver being able to adjust the dose by cutting the ODF to the size corresponding to the desired dose [60], and, unlike other ODx, it can be manufactured in a pharmacy setting as well [61].

The excipients used in the formulation of ODFs are film-forming polymers (hydroxypropyl methylcellulose (HPMC), hydroxypropyl cellulose (HPC), carboxymethyl cellulose, etc.) and plasticisers (glycerol, polyethylene glycol, propylene glycol), Among the film-forming excipients, some of the most popular are polyethylene oxide and Kollicoat^®^ IR, which is a mixture of polyvinyl alcohol (PVA) and polyethylene glycol (PEG) units [59,62].

HPMC, HEC, PVA and xanthan gum have been studied as film-forming excipients for a formulation containing meloxicam and tizanidine, along with croscarmellose, aspartame, PEG 400 and PVP K30. All the formulations exhibited similar dissolution profiles and increased bioavailability compared to the control [63]. Organoleptic excipients such as colouring and flavouring agents or sweeteners are added to the formulation to increase acceptability [59]. For taste-masking purposes, rupatadine has been enclosed in ethylcellulose microparticles through spray-drying. The resulting particles were suspended in a polymeric solution and cast into ODFs; those having performed well in the electronic tongue assessment [63,64].

The methods employed in the manufacturing of ODFs have evolved since their dawn in the 1960s. The first and currently most frequently used process is the solvent casting method [65], which, as the name suggests, involves dispersing the API and excipients in a solvent, then pouring the dispersion into a cast, sometimes lined with anti-adherent materials (polyester, polypropylene, Teflon^®^). After casting, the film is dried in an environment suitable for all the components [66]. The possibility of performing this process without the use of industrial-grade equipment makes it suitable for small-scale applications as well. As an alternative method, electrospinning, that involves the ejection of a thin stream of solution through an electricity-conducing nozzle onto a collecting drum, emerged. Polymer-drug mixtures can thus be turned into highly porous micro- or nanofibres with good wetting and disintegration properties [67]. For APIs that might change their properties when coming in contact with any solvents, but have acceptable heat stability, hot-melt extrusion could be selected for ODF preparation. This method entails heating the API-excipient mixture inside a barrel, extruding it through a die and finally, passing it through a series of cylinders to form a film. Since cellulose-based polymers are easily degraded at high temperatures, the excipients used in hot-melt extrusion must be carefully chosen, based on their behaviour when subjected to higher temperatures [59].

The more recent approaches in the preparations of ODFs are the inkjet printing of an API solution or suspension onto a fitting substrate, such as edible rice, sugar or starch paper, and the 3D printing of ODFs through extrusion techniques [59].

Although ODFs have been found to be highly acceptable even in neonates [68], their handling can be challenging even for the more dexterous of patients or caregivers. ODF monographs mention the need for sufficient mechanical strength to allow handling [69]; yet, that may differ depending on the formulation and manufacturing process [6]. For the ODF to become a more widespread dosage form, formulations must be optimised in a way that allows them to be easily transported, handled and fractioned to the appropriate dose.

### 2.6. Shortcomings of ODx

Each orodispersible dosage form has its own advantages and disadvantages, but most drawbacks of these products are common to all of them, so these will be summarised in the following section.

Most ODx exhibit low tensile strength, either due to the low compression force used in the manufacturing of ODTs [12] or due to the nature of excipients and processes used in the manufacturing of OLs [70] and ODFs [6].

Poor oral bioavailability caused by reduced water solubility does not affect only APIs included in ODTs, but in all ODx; while the orodispersible factor aids the API dissolution, there might still be problems in achieving the desired bioavailability due to the API’s low solubility and/or permeability, including through pre-gastric mucosa [6]. These problems could be approached through the use of nanostructures, either as nanofibres or nanocrystals that could be used as easily disintegrating compression material or formulated into ODFs or OLs with enhanced API dissolution [34,71,72].

Furthermore, if the API is unloaded from the dosage form directly into the oral cavity, as it happens in the case of ODx, regardless of its absorption site, achieving a particular release profile is impossible. To avoid this obstacle, the API must be included in a coating or another structure that allows it to resist the acidic conditions in the gastric cavity or is able to release it at a certain pace [13].

Given that the ODx are the dosage forms that grant the longest contact time between the API and the tongue, masking the unpleasant taste of the API is of paramount importance. Although ODTs and OLs frequently include sugars and sugar alcohols as both structural and organoleptic excipients [12,20], their taste-masking capabilities might not be enough at the moment of the disintegration of the dosage form. At the same time, these excipients are less common in ODF formulations, so these are in an even more dire need of other ways of taste-masking.

In terms of the ODx intended for administration to paediatric patients, an additional concern regarding their safety appears due to the potential adverse effects inflicted by certain excipients [5].

This is where polymeric and lipidic nanostructures come into play, by being able to coat the API in a layer that can mask its taste, enhance its bioavailability and delay its release, while also being highly biocompatible [73,74].

### 2.7. Quality Profile of ODx for Paediatric Use

Orodispersible dosage forms are rather overlooked in terms of characterisation, with both the European and the United States’ pharmacopoeias having concise monographs for ODx. In the case of ODTs and OLs, these include the definition of the dosage form and at most two tests that can be performed as quality control specific to the product. The tests are lacking altogether in monographs for forms such as ODGs and minitablets, both orodispersible and not. While some tests from larger general monographs, such as the mechanical resistance of tablets, can be adapted to ODx, there is a need for individualised tests for the specific dosage form. Appropriate quantification methods are necessary for certain dosage form characteristics, such as the disintegration time of ODFs, to create a uniformised Quality Target Product Profile (QTPP) that comes to benefit developers and manufacturers, as well as patients.

The QTPP, implemented by the International Council for Harmonisation (ICH), is the foundation of a pharmaceutical development process, and a summary of the quality attributes the final product would ideally embody, including characteristics such as dosage strengths and release profile [75,76]. Aside from the regular QTPP, a particular type, the paediatric Quality Target Product Profile (pQTPP), has been conceived. The pQTPP is necessary for developers and formulators to be able to tailor medicines either specifically to the needs of different age groups, or flexibly, in a way that allows the administration to a larger age bracket, as well as creating a uniformised quality profile for future medicines. Moreover, compared to a classic QTPP, it includes characteristics that have a higher impact on the paediatric patient and the efficacy and safety of their treatment, such as palatability. Walsh et al. have developed a model pQTPP, along with a risk score-based tool previously conceptualised by the WHO, to identify whether the medicines in the pre-development or development phase, as well as the already existing products containing APIs included in the Model List of Essential Medicines for Children, are fit for paediatric pharmacotherapy [77].

QTPPs have appeared throughout the specialised literature for multiple dosage forms, including orodispersible and nanoparticulate-based ones. The ODx QTPPs contain the usual attributes included in the quality profiles of solid oral dosage forms, such as the dosage form, administration route, appearance, dosage strength, assays, pharmaceutical properties, stability and microbial load that need to be met and particularised to the orodispersible dosage form, such as the package attributes or the peel adhesion, in the case of ODFs [25,59,78,79]. However, the lack of proper pharmacopeial characterisation makes the development of a QTPP more challenging. Certain acceptance limits ought to be modified to suit the dosage form, namely the disintegration time and dissolution profile, that should be in concordance with the European Pharmacopoeia or FDA requirements [8,9], while others have not been officially declared. This is the case of tensile strength, whose values often vary from one study to another, usually keeping an inferior limit that allows the manipulation of the dosage form throughout its life cycle, from manufacturing to administration [25,59,78,79]. For these QTPPs to fit the paediatric criteria, some other characteristics need to be included, such as the target population, dose flexibility and/or banding, patient acceptability, excipient safety, the manipulation required before administration and the ease of providing the patient with a correct dose upon every administration [77,80].

Out of the European Pharmacopoeia monographs [8,58], several research articles in the fields of orodispersible dosage form development [25,55,59,78,79,81,82] and paediatric formulations [70,77,80], respectively, have led to the development of a model QTPP for ODTs, presented in Table 1.

Along with the dosage strengths, that need to be tailored to the patients’ age and developmental stage or banded for different age categories, there is one more attribute that may vary between different age groups within the paediatric population: the exact dosage form that would be appropriate for the patient, while also being highly acceptable, as to ensure the treatment adherence. Even though it is obvious that this is subject to interindividual variability, there are some patterns in terms of dosage form preference, highlighted in several studies, either performed in clinical settings or based on surveys given to the paediatric patients or their caregivers.

Several recent investigations present an overview of a vast population’s response to a wide range of dosage forms, frequently involving multiple European countries [83,84]. In terms of ODx, the information revealed is somewhat limited due to the rather scarce presence of these dosage forms on the market compared to other oral paediatric dosage forms. A factor to consider as a cause of the less frequent usage of ODx could be the higher cost of commercially available ODx compared to their more common counterparts, liquid formulations and tablets. These shortcomings, when not accounted for in a study environment, could falsely indicate low patient acceptability.

Small-scale research results can also be used to guide dosage form selection in different age groups. These studies usually target children’s reactions to either two dosage forms containing the same API, the API-loaded compared to the placebo product or they could be run on just one dosage form [68,85,86,87,88,89,90,91,92]. A strategy for dosage form selection based on patients’ ages was proposed by Mistry et al. [93]. Some small-scale studies assessing the preferences for ODx have been summarised in Table 2.

Following the selection of an appropriate ODx, its critical quality attributes (CQAs) may be defined, such as disintegration time, tensile strength and friability for ODTs (Table 1). Supplementary CQAs could be added to each ODx according to its special features like folding endurance or peel adhesion for ODFs. Among the pharmaceutical characteristics, disintegration is of paramount importance for all ODx, with important implications for other features. Although official pharmacopoeial tests are non-specific, the literature abounds in methods intended to mimic the oral disintegration of the ODx, and the mechanical manipulation they suffer. Most of the methods rely on a texture analysis [94,95,96] or imaging techniques [97], and the results were summarised in several reviews [6,98].

Upon disintegration, the API is immediately swallowed, and in some cases, a part of the dose might be absorbed through the sublingual mucosa. While this comes as an aid when the rapid onset of action is needed, certain APIs need temporal control over drug release (sustained release, extended release, delayed release, etc.). Coating or encapsulating API particles in a modified-release or gastro-resistant polymer could solve more problems at once: protect the API from acidic media, modify the release and mask the API taste [13].

Moreover, disintegration in the oral cavity entails direct contact between the gustative receptors and the API, which often exhibits an unpleasant taste with an impact on patient acceptability and treatment adherence. Therefore, taste-masking techniques should be used as part of the formulation process of ODx. Among those, the most common is the use of sweeteners and flavourings in the formulation, but processes such as API complexation/derivatisation, or coating/encapsulation can grant a more effective and safe taste-masking, especially when considering the adverse effects that can come with the administration of certain organoleptic excipients [99,100]. As a response to the need for taste assessment, instrumental methods were developed in the form of electronic tongues based on potentiometric or impedance spectroscopy sensors [101], or on the foundation of ultraviolet spectroscopy [6,98,102].

The palatability of ODx is one of the toughest characteristics to assess since it extends past the simplistic fields of taste and flavour into traits such as texture and mouthfeel. While an in vivo assessment can be performed in the form of a patient- or volunteer-based tasting panel, in which the subjects usually rate the taste and mouthfeel perception on a hedonic scale [94,96,103,104], the results are heavily impacted by interindividual variation and subjectivity of the study participants. Hence, instrumental methods to assess the palatability of ODx are a necessity for the creation of a proper QTPP. Regarding the mouthfeel of the dosage form, a correlation between the tribological characteristics of the dosage form (studied through texture analysis) and the responses received from a human tasting panel has been developed for both ODTs and regular coated tablets [94,95]. However, a texture analysis does not provide any information regarding the taste profile, so it could be used as a complementary technique to the electronic tongue system [101,105,106,107].

In the case of formulating an ODx in which the API is included as a nanosystem, nanosystem-specific quality attributes are required, for example, the zeta potential, particle size and polydispersity index, along with an indication of the purpose the nanosystem serves: increased acceptability due to taste-masking, an improved pharmacokinetic profile [108,109] or better stability compared to other dosage forms with the same API [110,111]. ODx, while promising as dosage forms for the paediatric population and others, are still insufficiently investigated in terms of their formulation aspects. Hence, they are more difficult to particularise for APIs with decreased solubility and/or permeability and with an unpleasant taste, with the latter being a matter of high concern in the oral administration of medicines to children. At the same time, there are several concerns regarding the excipients used in formulations intended for paediatric use, because of the potential adverse effects caused by excipients, including taste-improving substances.

## 3. Nanotechnology-Based ODx

### 3.1. Nanostructures in Oral Drug Delivery

The use of nanotechnologies can be a solution to multiple shortcomings encountered in the formulation process of orodispersible dosage forms, especially those addressed to the paediatric population. The main shortcomings are related to the solubility and permeability of APIs, which can impede, one way or another, the absorption, distribution or pharmacological effect of the drug. These can be approached through the use of nanostructures, obtained either through particle size reduction to the nanoscale range (nanonisation) of the API or through its inclusion in a nanocarrier, functionalised in a way that allows the dissolution and permeation to take place. The first approach involves the preparation of nanocrystals, while the second most commonly involves the use of polymeric and lipid-based nanocarriers [112,113].

Nanocrystals can be the result of a top-down approach, i.e., the milling of the API powder down to the desired particle size, or a bottom-up process, consisting of dissolving it into an appropriate solvent and subsequently using the antisolvent precipitation method under continuous stirring [108,110].

Regarding the preparation of nanocarriers, the top-down methods are similar to those used for the preparation of nanocrystals, involving the particle size reduction of the bulk material through various processes: milling, sputtering, thermal evaporation or laser ablation. After obtaining a product with the expected particle size, it may be coated with a polymer for stability or functional purposes. The bottom-up approaches can become more sophisticated, however, often involving the use of the solubility profile of substances in specific solvents [114,115,116]. The most commonly used method in the manufacturing of protein or polymer-based nanostructures, antisolvent precipitation [74,117], goes in this category, along with lyophilisation [118], ionotropic gelation [119], chemical vapour deposition, hydrothermal synthesis and the sol-gel method [116].

Orodispersible dosage forms, having a longer oral residence time compared to other dosage forms, require the use of several excipients to ensure a pleasant taste profile. However, what has not been taken into account by formulators for a long time is the idiosyncrasies of the paediatric population in relationship not only with the APIs used in therapy, but with certain excipients, which can trigger adverse effects, especially in younger children [120]. Due to their high biocompatibility, lipid and polymer-based nanostructures can be administered with fewer negative effects inflicted by excipients, while also enhancing acceptability through the coating or inclusion of the unpleasant-tasting API within said polymers or lipids [100].

The reduced size of nanostructures grants an increased contact area, enhancing the water solubility of drugs belonging to the second and fourth classes of the Biopharmaceutical Classification System (BCS), hence allowing for the administration of smaller doses. Since the drug bioavailability is increased, the administered doses can be lowered, hence improving the safety profile [118].

Through the variety of raw materials that can be used, polymeric nanocarriers can address the poor absorption or distribution of the API upon oral administration or help turn ODx into modified release dosage forms.

Regarding the polymer-based structures, the APIs can be incorporated into nanoparticles or nanofibres [72,121]. Several polymers frequently used in the development of nanostructures, including poly(lactic acid) (PLA), poly(lactic-co-glycolic) acid (PLGA) or polystyrene can enhance a drug’s absorption upon oral administration by increasing the intestinal uptake of the encapsulated molecules, while also being able to form a sustained-release matrix and protect the drug from the harsh conditions in the gastrointestinal tract. In addition, PLGA, PEG or chitosan, used either as matrix-forming agents or as coatings applied after the formation of the nanostructure, can improve absorption by adhering to the intestinal mucus layer or binding to enterocytes or M cells. Besides the use of mentioned polymers, targeting M cells may be specifically achieved by way of lectins: a group of proteins and glycoproteins of plant or bacterial origin, grafted onto the nanoparticle surface [113]. Functional polymers, such as acrylic and methacrylic esters, can also be used either in the main structure of the nanoparticle [122] or as a coating applied after their initial formation [123]. In this manner, nanostructures can be customised according to the API’s particularities to maximise treatment efficacy, while also ensuring taste-masking. In this respect, both polymeric [74,123,124,125] and lipidic [100] nanostructures can be employed.

Nanocarriers can bypass the P glycoprotein (P-gp) multidrug resistance pump, both because of their size (under 500 nm) and through the inclusion of P-gp inhibitors in their structure or in the dosage form. Among the P-gp inhibitors, there are some common excipients, such as TPGS, Tween, polymers (chitosan, natural gums, Poloxamers) or phospholipids [126].

Lipid-based nanoparticles have been found to be able to bypass first-pass metabolism by allowing absorption through the lymphatic system, the way through which the majority of a lipid nanocarrier-contained drug enters the systemic circulation [127,128]. This behaviour is exhibited by several types of lipid-based nanocarriers, including but not limited to solid lipid nanoparticles (SLN), phytosomes, liposomes, nanostructured lipid carriers (NLC) and self-nanoemulsifying drug delivery systems (SNEDDS) [129,130].

Liposomes, some of the most frequently employed nanostructures in pharmaceutical formulations, can be used in oral drug delivery, although their nature makes them susceptible to degradation in the gastrointestinal tract. Due to the elasticity added by the edge activator, transfersomes can enhance drug permeation through entering pores and channels that are not usually accessible for other types of nanocarriers [131].

One particularly enticing potential use of nanostructure-containing orodispersible formulations is the administration of drugs meant to be absorbed through the buccal mucosa, which can be useful for the immediate onset of action of APIs as well as in enabling oral administration of peptides and proteins, currently incompatible with oral administration. This could be achieved by combining certain nanostructures with substances such as permeation enhancers and inhibitors of the enzymes present in saliva, most specifically protease, although that could be adapted depending on the nanostructure [132,133].

### 3.2. Benefits of the Use of Nanostructures in ODx

Given the multiple applications of nanostructures in the manufacturing of oral dosage forms and the number of patients that could benefit from them, there have been several attempts to build API-based nanostructures into orodispersible preparations. These will be presented further, according to the shortcomings they address as the main target of the proposed formulation. It is to be noted that not all the APIs in the following formulations are used on a large scale in paediatric pharmacotherapy, although all of them are relevant from a technical viewpoint since the methods could be adapted to accommodate other active substances.

Since the bioavailability of APIs is one of the crucial aspects in the formulation of orally administered dosage forms, it is the most frequently sought-after improvement in the development of nanostructures designed for this administration route. For this purpose, both polymeric/protein-based [72,134,135,136,137,138,139] and lipidic nanostructures [131,140] have been employed and included in ODx.

The most frequently encountered approach is the processing of the API into nanostructures in order to improve its pharmacokinetic properties, be it through improving the water solubility or other means. For increasing the water solubility and/or dissolution rate of APIs, a frequently used approach is the use of polymers, either through forming nanocarriers [34,55,73,139,141,142,143,144,145,146,147,148,149] or using them to stabilise powder particles that were milled and dispersed, so as to avoid their aggregation [143,144,146]. Steiner et al. performed the latter technique in the form of stirred media milling to obtain suspensions of naproxen and anthraquinone (as model substances) that were subsequently formulated into ODFs, and an instant ODF formulation that can be reconstituted in water prior to administration. Both manufacturing processes followed the same steps up to the preparation of the film casting mass, after which the process diverged according to the target product: while ODFs followed the classic procedure of solvent casting, the instant ODF formulation was subjected to spray-drying to be properly preserved until the moment of reconstitution [143,144].

Similarly, aripiprazole has been co-processed with poloxamer 407 through ball-milling into a solid dispersion that exhibited a hundred-fold increase in API solubility compared to the pure substance. The resulting product was casted into orodispersible films with acceptable physical properties, in which the solid dispersion also allowed for an accelerated disintegration [146].

Nitrendipine has been prepared into a nanosuspension by way of antisolvent sonoprecipitation. Upon the completion of the precipitation process, the nanosuspension was treated in two different ways: the formation of nanocrystals through lyophilisation with the purpose of being compressed into an ODT, and the inclusion of film-forming excipients in the suspension prior to casting an ODF, respectively [141,142].

Another method of achieving the higher water solubility of an API was by the inclusion of the API in vitamin E-TPGS and Soluplus^®^ nanomicelles obtained through the film hydration method; the final product proving itself to enhance the solubility and permeability of aripiprazole following oral administration [150], with the possibility of being included into an ODx in the future. Herpetrione, an antiviral agent of vegetal origin, was turned into a nanosuspension with povidone K30 and sodium dodecyl sulphate as stabilising agents for the improvement of its water solubility and further formulated into an orodispersible film for the ease of administration [145].

Nanostructures have been used in orodispersible formulations that exhibited a certain release profile [119] or that also included unencapsulated API alongside the nanostructures, achieving a dual release profile: first, the free API was released in the oral cavity for the rapid onset of action, then the nanocarriers disintegrated in the gastrointestinal tract for a prolonged effect [73,139]. In an attempt to achieve a sustained release system for scopolamine, a cross-linked chitosan nanoparticle suspension was spray-dried in order to obtain micronic aggregates, which were subsequently compressed into orodispersible minitablets [151].

Studies on the inclusion of API nanostructures in ODx for their taste-masking and paediatric acceptability-increasing capacities have been performed, with both encapsulation and electrospinning having proved to be successful in concealing the unpleasant taste of different active substances [74,119,125,136,138,146].

Prednisolone is a corticosteroid drug frequently administered to children, and hence, an orodispersible tablet is already commercially available. However, Tawfik et al. attempted to manufacture PVA-based electrospun nanofibres and free drug-containing ODFs to assess the taste-masking capabilities of these processes. Given the bitter taste of prednisolone and the much longer disintegration time the films needed compared to the nanofibres, the electrospinning method was deemed as the most appropriate for further studies [136].

Antiretrovirals constitute a class of medications that in certain occasions need to be administered chronically from the earliest stages of a child’s life; hence, there is a need for dosage forms containing extremely low doses. At the same time, the medicine has to be safe and easily swallowable, while also avoiding the spitting reflex of infants as much as possible. To address this, Deng et al. developed a formulation based on lopinavir and ritonavir nanoparticles coated in Eudragit^®^ E PO that performed well in the electronic tongue test and surpassed already existing solid oral formulations in terms of the in vitro dissolution profile and the in vivo plasmatic concentration when administered to rats [74]. Since tuberculosis is still a matter of concern in underdeveloped countries, there is a need for dosage forms of antitubercular medications that can be safely administered to children, while also ensuring high treatment adherence for efficient prophylaxis. For this purpose, an isoniazid ODF based on electrospun nanofibres made of pullulan associated with HPMC, casein or pectin has been developed. The HPMC formulation was the most suitable for further studies, being characterised by its fast disintegration and drug release, that could ensure proper absorption within the oral cavity. While this study proved the possibility of nanofibre electrospinning from natural or semisynthetic materials with lower risks regarding patient safety, further investigations regarding taste-masking are needed [125].

### 3.3. Preparation of ODx Formulations with Nanostructures

Some of the processes involved in the manufacturing of nanostructure-based ODx do not stray far from the ones applied to unmodified APIs, as is the case of compression [119] or solvent casting [143]. A process such as electrospinning may create the nanostructure already encased in what will become the dosage form, as happens in the case of ODFs [72], but may as well create an intermediate product that will be further processed, e.g., compressed [34]. In the case of excipient safety-related concerns, electrospinning is a suitable approach for using as few excipients as possible, with nanofibre-based formulations often including solely the API and the polymer used in the fibre matrix [72,125,136,138] or a reduced number of excipients [34].

Several published examples of the methods employed in the manufacturing of nanostructure-based ODx, along with details on formulation and nanoparticle preparation processes, can be found in Table 3.

ODTs containing nanostructures were prepared via direct compression using common fillers such as MCC, mannitol, dextrates or lactose [34,119,139,141]. When incorporating electrospun nanofibres into ODTs, they were initially pulverised in the presence of a diluent to decrease particle size and facilitate homogenisation. The success of the size reduction process depended on the nanofibre polymer, with better performance for Eudragit E when compared to PVP K30. Eudragit E nanofibres also led to ODTs with faster disintegration than PVP K30 explained through the lower gelling tendency of Eudragit. Therefore, the nanofibre composition had an important impact on downstream processability and on solid disintegration [34].

Nitrendipine nanocrystals obtained through antisolvent precipitation and lyophilisation were mixed with MCC, mannitol, lactose and croscarmellose and compressed, resulting in ODTs with an improved in vitro release compared to a commercially available tablet [142].

Coated and uncoated chitosan nanoparticles were also directly compressed into ODTs and displayed no negative impact either on the tensile strength of tablets nor on their disintegration time [139]. In another formulation, scopolamine microaggregates obtained from a spray-dried cross-linked nanoparticle suspension went through wet granulation with a PVP K30 solution. The resulting granules were mixed with MCC, carboxymethyl starch sodium, mannitol, lactose, xylitol and magnesium stearate, then the mixture was compressed into ODTs. When compared with ODTs containing either the free API or aggregates built from non-cross-linked nanoparticles, the novel formulation showed an improved sustained release profile [151].

A particularly interesting approach, with significant potential for use in orodispersible dosage forms, is the formulation of tablets containing pre-liposome powder, in which the liposomes form in situ upon the wetting of the tablet, providing the advantages of these nanostructures while maintaining a manufacturing process as easy and reproducible as possible (Vanic et al. [152]).

Another perspective towards the use of nanomaterials in the manufacturing process of orally disintegrating dosage forms is their potential use as excipients. Cellulose nanofibres have been used as co-diluents with lactose in a directly compressible paracetamol orodispersible tablet formulation: the final product exhibiting elevated hardness, comparable to that of microcrystalline cellulose, and a swift disintegration [153]. When higher compression forces (≥10 kN) were applied, the friability was under 1%, making the resulting tablets compliant to the European Pharmacopoeia requirements [153,154]. This requirement was met due to the compression behaviour of the cellulose nanofibres, which released even smaller particles that allowed the formation of a dense outer layer of the tablet [153].

While the inclusion of nanostructures into ODTs requires either drying or milling phases, OL and ODF preparation relies on the use of nanosuspensions, which can become the base in which the excipients needed for solvent casting or freeze-drying are dispersed, with neither of those processes risking the infliction of any damage on the nanostructures. For this reason, lyophilisation is often used as a way to increase the stability and shelf life of nanostructure-based drug products [155].

**Table 3:** Studies regarding the inclusion of nanostructures in ODx formulations.

ODx	ODx Formulation	API	Nanosystem/Preparation Method	Nanosystem Formulation	Benefits of the Inclusion of Nanosystems	Reference
ODTs	Filler: MCC, mannitol SD: croscarmellose sodium	Meloxicam	Nanofibres/electrospinning	Eudragit^®^ E, PVP K30	Improved API dissolution	[34]
	Filler: MCC, lactose, mannitol SD: croscarmellose sodium Lubricant: magnesium stearate	Nitrendipine	Nanocrystals/antisolvent sonoprecipitation followed by freeze-drying	Stabiliser: HPMC E6	Faster and complete release of API Improved oral bioavailability in rabbits	[142]
	Filler: dextrates, silicified MCC SD: sodium starch glycolate Sweetener: sodium saccharine Lubricant: sodium stearyl fumarate	Meclizine	Polymeric nanoparticles/antisolvent precipitation	Polymers: chitosan, shellac Stabiliser: Poloxamer 188	Dual function compressed tablet whose core contained nanoparticles with prolonged release, while the outer layer contained the soluble free form for buccal absorption Higher bioavailability of API from novel formulation compared to commercial product	[139]
	Filler: lactose SD: L-HPC Lubricant: magnesium stearate	Promethazine	Polymer-coated nanoparticles/ionotropic gelation	Polymer: chitosan Coatings: PEG, PVP, polyethylene co-acrylic acid	Sustained release of the API; the API release decreased: ODTs free API > ODTs noncoated NP > ODTs coated NP	[119]
	Filler: MCC, mannitol, lactose SD: sodium starch glycolate Sweetener, diluent: xylitol Lubricant: magnesium stearate	Scopolamine hydrobromide	Polymeric nanoparticles/ionotropic gelation	Polymer: chitosan Cross-linker: tripolyphosphate	Improved sustained release profile when compared to formulations containing non-cross-linked polymer systems or free API	[151]
ODMTs	-	Prednisone	Nanofibres/electrospinning	PVP	Increased solubility compared to the API powder	[138]
OLs	Filler: mannitol MFA: sodium alginate, sodium croscarmellose	Meloxicam	Nanocrystals/high pressure homogenisation	Stabiliser: Poloxamer 188	Faster API dissolution	[55]
	Filler: maltodextrin MFA: xanthan gum/croscarmellose/gelatin Co-binder: PEG 4000	Piroxicam	Nanocrystals/high pressure homogenisation	Stabiliser: Poloxamer 188	Improvement of API dissolution rate Improved dissolution profile compared to commercial product	[147,148]
	Filler: sucrose MFA, viscosity agent: PVA Co-binder: PEG 6000	Silymarin	Mesoporous silica nanospheres/solvent evaporation method	Mesoporous material formers: Hexadecyltrimethyl ammonium chloride, tetraethyl orthosilicate	API fast dissolution rate, high saturation solubility	[149]
	MFA: Pullulan Binder: HPMC Sweetener: aspartame, xylitol SD: Plasdone XL	Rosuvastatin	Transfersomes/lipid film hydration	Phospholipid: soybean phosphatidylcholine Edge activator: Tween 80 Negative charge inducing agent: dicetyl phosphate	Superior pharmacokinetic and pharmacodynamic performance compared to commercial drug	[131]
ODFs	FFA: HPMC Plasticizer: glycerol	Naproxen, anthraquinone	Polymer-coated nanoparticles/stirred media milling	Stabiliser: vinylpyrrolidone–vinyl acetate copolymer, sodium dodecyl sulphate	Improved API dissolution	[143,144]
	FFA: HPMC E6, PVA SD: croscarmellose Plasticizer: PEG 400	Nitrendipine	Nanocrystals/antisolvent sonoprecipitation	Stabiliser: HPMC E6	Improved API solubility API in the amorphous state	[141]
	FFA: HPMC SD: L-HPC Filler: MCC Sweetener: mannitol Plasticizer: PEG–400	Herpetrione	Polymer-coated nanoparticles/high pressure homogenisation	Stabiliser: povidone K30 and sodium dodecyl sulphate	Improved API dissolution API in the amorphous state	[145]
	FFA: Kollicoat IR/Kollicoat Pr/PVA Plasticizer: glycerol	Aripirazole	Nanoaggregates/high energy ball milling	Stabiliser: Poloxamer 407	100-fold improvement in API solubility when compared to raw API	[146]
	FFA: PVA Plasticizer: PEG 1500	*Vaccinium arctostaphyllos* extract	Liposomes/lipid film hydration	Soybean phosphatidylcholine	Better active ingredient release from liposome formulation compared to the pure extract formulation	[140]
	FFA: guar gum Sweetener: sorbitol Penetration enhancer: citric acid	Alpha-casozepine	Polymeric nanoparticles/antisolvent sonoprecipitation	PLGA	Enhancement of buccal and intestinal permeation compared to the API dispersion Protection of the API along the GI tract	[156]
	-	Amlodipine	Nanofibres/electrospinning	Carboxymethylated curdlan, poly(ethylene oxide)	Faster onset of action, improved API absorption and bioavailability compared to the commercial product	[72]
	FFA: HPMC E15, PVA, maltodextrin Plasticizer: PEG 600, glycerol Sweetener: mannitol, aspartame	Dimethyl fumarate	Polymeric nanoparticles/ionotropic gelation	Core: sodium alginate Shell: chitosan	The formulation allowed dose reduction and enhanced bioavailability compared to pure drug	[134]
	-	Prednisolone	Nanofibres/electrospinning	PVA	Faster disintegration and drug release when incorporated into fibres compared to the solvent casted film	[136]
	FFA: HPMC E5/E15, PVA Plasticizer: PEG 400, TPGS 1000	Lercanidipine	Nanosuspension/antisolvent precipitation	Stabiliser: PEG, Hypromellose, PVA, Alginate, TPGS, HPC, Methylcellulose	Increased API solubility when compared to the free drug	[137]
	-	Isoniazid	Nanofibres/electrospinning	Pullulan + Hypromellose/pectin/sodium caseinate	The pullulan/Hypromellose films disintegrated within 5 s and released the API content within 30 s	[125]
	FFA: HPMC E15, PVA Plasticizer: propylene glycol	Buspirone	Polymeric nanoparticles/antisolvent precipitation	Polymer: PLGA Stabiliser: Poloxamer 188	Dual release: fast release of the free form and sustained release of API from nanoparticles	[73]
	-	Valsartan	Nanofibres/electrospinning	PVP K90	Enhanced API release compared to the physical mixture or free drug	[157]
	-	Lopinavir and ritonavir	Polymer-coated nanoparticles/antisolvent precipitation	Coating: Eudragit^®^ E PO	Taste-masking of the APIs has been proved with the aid of the electronic tongue	[74]

ODx—orodispersible dosage form; ODF—orodispersible film; ODT—orodispersible tablet; OL—oral lyophilisate; ODMT—orodispersible minitablet; MFA—matrix-forming agent; FFA—film-forming agent; SD—superdisintegrant; HPMC—hydroxypropyl methylcellulose; HPC—hydroxypropyl cellulose; L-HPC—low-substituted hydroxypropyl cellulose; PVA—polyvinyl alcohol; PEG—polyethylene glycol; MCC—microcrystalline cellulose; PVP—polyvinylpyrrolidone; TPGS—tocopheryl polyethylene glycol succinate.

Mesoporous silica nanoparticles containing silymarin were included in an OL formulation with PVA as a precipitation inhibitor and binder, and sucrose as a cryoprotectant and stabiliser, with the dosage form proving itself to be sufficiently physically resistant besides improving the dissolution rate of the API [149]. Good results have also been achieved in the case of OLs made from a piroxicam nanosuspension stabilised with Poloxamer, to which PEG 4000, maltodextrins and various matrix formers (xanthan gum, gelatin, croscarmellose) have been added. All of the proposed formulations had a greater API dissolution rate compared to OLs prepared from a coarse suspension, while the gelatin and croscarmellose OLs performed better than the commercially available piroxicam OL, nonetheless proving the beneficial effect of nanonisation [148]. Pullulan-based OLs with HPMC, aspartame, xylitol and Plasdone XL that include rosuvastatin-loaded transfersomes elevated the plasma concentration of the API when compared to a commercial product, while maintaining a similar release profile [131].

In the case of ODFs, the most frequently used manufacturing approaches are solvent casting and electrospinning. Solvent casting needs to be applied on a previously prepared nanosuspension in which the film-forming and plasticising excipients are dissolved. An innovative concept in the field of solvent-casted ODFs is the instant ODF, a formulation based on spray-drying the casting dispersion for it to be redispersed in water before administration. A team of researchers developed a casting dispersion containing naproxen and antraquinone nanoparticles stabilised with Kollidon VA and sodium dodecyl sulphate obtained through stirred media milling. After this process, the HPMC (film-forming agent) and glycerol (plasticiser) were dispersed in the nanosuspension, with this aqueous dispersion being subsequently treated in two ways: classic solvent casting and spray-drying; the latter providing the redispersible product. While both approaches were successful in terms of the physical properties of the dosage form and an increased API dissolution rate, the interaction between the nanostructures and the film-forming polymer still needed to be studied [143,144].

Aside from the research concerning the formulation of certain APIs into nanostructure-based ODx, studies on the compatibility of nanoparticles with the process of being formulated into orodispersible films have been performed and proved successful for both solid lipid nanoparticles (Steiner [158]) and polymer-based systems containing anthraquinone as a model substance (Steiner [159]). As for the effect of the nanostructures on the dosage form, it has been demonstrated that PLGA nanocarriers formulated into an ODF increase its adhesion to the tongue upon administration [156].

There is a lack of studies regarding the way certain processes, such as compression, may impact the nanostructures in an oral dosage form, or vice versa, the way the existence of a certain nanostructure in the powder mixture intended for tableting affects the compression process, which might be an interesting research direction to approach in the future. However, the amount of works describing successful attempts at formulating nanostructure-based ODx presented in this review article stands as evidence that these dosage forms can be manufactured and have an appropriate quality profile [34,72,125,136,138,156,158,159].

## 4. Paediatric Orodispersible vs. Nanostructure-Containing Orodispersible Forms: Current Research Landscape and Commercially Available Products

Since the introduction of regulations addressing the necessity for specific paediatric dosage forms in the European Union and the United States, researchers slowly started making room for this particular field among their interests and priorities. Hence, after the year 2007, when the aforementioned regulations came into effect, publications regarding the formulation and characterisation of orodispersible dosage forms became more and more frequent. Inevitably, the ever-growing field of nanotechnologies transpired into the pharmaceutical industry as well, with successful and already marketed results for injectable medications. In the past years, there has been an interest in developing nanostructure-based oral dosage forms, especially paediatric and orodispersible dosage forms, since certain nanostructures can solve the most common shortcomings encountered in the formulation process of these products.

At the beginning of April 2022, an article search was performed in the Web of Science (WoS) and Scopus databases, with the following queries: “orodispersible dosage forms paediatric”; “orodispersible dosage forms nano* paediatric”; “orally disintegrating dosage forms paediatric”; “orally disintegrating dosage forms nano* paediatric”, including their respective versions with the word “pediatric” as well.

**Figure 2:**
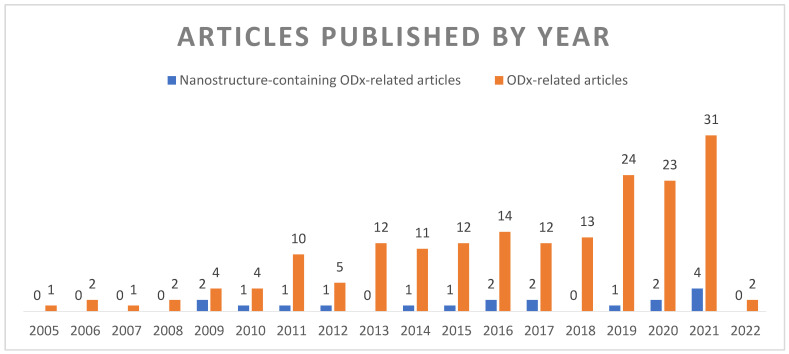
Trends in ODx and nanostructure-based ODx research.

All the queries used on WoS were used on Scopus as well, but the results of two of those searches were identical to the results of the other two and were removed for ease of processing. The search results were imported into EndNote for duplicate identification and sorting by year. Afterwards, the references were organised by year in Microsoft Excel; a graph showing the research trends has been generated and is presented in Figure 2.

This visual suggests that research teams have exponentially gained interest in the development of orodispersible dosage forms for paediatric use in the past years. However, the development of nanostructures for this purpose seems to be taking off much slower, most probably due to the large interest in their other uses.

To understand the need for more intense research in the field of paediatric medications, an ensemble view of the current situation is needed. A non-exhaustive search has been performed among the medicines authorised for paediatric use in the European Union, which are orodispersible products. Thus, the Mutual Recognition Information (MRI) product index of the Heads of Medicines Agencies was used to identify the products approved in the Member States of the European Union according to the Mutual Recognition or Decentralised Procedure [160] and the European Medicines Agency human medicines database for the products approved through the centralised procedure [161]. The identification of the products was followed by the analysis of their summaries of product characteristics (SPCs) found either on the EMA website or on the websites of the respective national agencies that authorised said products. The APIs contained by orodispersible products authorised for paediatric use (having been approved for usage from the age of 13 or less) that were identified during this process and some of their brand names can be seen in Table 4. The actual number of orodispersible dosage forms available in the EU is much larger, although most of them do not have the clinical studies needed to prove their safety and efficacy in the case of administration to children or are not used in paediatric pharmacotherapy, as is the case of most antipsychotics and drugs addressed to patients with Parkinson’s or Alzheimer’s disease.

## 5. Future Perspectives

Although research in the field of nanostructure-based orodispersible formulations has started taking off in recent years, there are certain gaps in these studies. Along with studies regarding the improved efficacy and safety of the API, there is also a need for studies regarding the manufacturing aspects of these dosage forms, such as the effects of compression or other processes on the integrity of the nanostructures; once this effect is recorded, the focus can shift onto process optimisation. It would be equally useful to look into the way the existence of a certain nanostructure in a mixture of excipients may affect the manufacturing process, e.g., through a dynamic compression analysis. This gap reflects in the already existing research as well, given that the most encountered dosage form including nanostructures is the orodispersible film, followed by the oral lyophilisate, both of those being results of low-impact manufacturing processes. Still, while there are studies on the ways nanostructures may modify the quality profile of an ODF, it might be useful to look into the ways they affect OLs, which are known for their friability.

Another aspect that needs to be researched more in-depth is enhancing the drug loading of the nanostructures used in these formulations, since the encapsulation efficiency can be low in some cases. Even though they are orodispersible formulations, the larger sizes would result in prolonged disintegration times and would pose certain problems with paediatric patients, both in terms of safety, due to the risk of choking, and acceptability, because the patient may refuse to take the medication or be tempted to chew it, deteriorating the nanostructures in the process.

As nanostructures will become more widely used in oral dosage forms, especially for paediatric use, and more types of nanostructures will be considered for use, there will be a need for studies on the safety of the materials used in nanostructure formulations, regardless of their supposed biocompatibility.

## 6. Conclusions

Orodispersible dosage forms have the potential to elevate the efficacy, acceptability and safety of paediatric pharmacotherapy through their patient-friendly nature. However, their development has been hindered, among others, by several inconveniences that could not have been addressed through conventional formulation techniques, including the pharmacokinetic and biopharmaceutical particularities of the active substances and their organoleptic profile. Even though nanostructures have been a matter of interest predominantly as injectable drug carriers in the therapy of neoplasms, the plethora of materials that can be used in their preparation can enable their use in oral pharmacotherapy, especially since several shortcomings of oral and orodispersible medication can be easily overcome through their incorporation in different nanostructures.

## Figures and Tables

**Table 1:** Model QTPP for orodispersible tablets.

Quality Attribute			Target	Justification
Route of administration			Oral	Oral administration provides higher treatment compliance, especially in paediatric patients.
Patient age range			Pre-term neonates to 18 years; may be restricted to smaller groups depending on age or weight	Different dosage forms may be designed for different age groups and/or different dosage strengths.
Dosage form			ODT	ODTs are easily acceptable and can be safely administered to younger patients.
Dose			According to the API and target population	Dose increments/banding may be particularised according to the target age or weight range.
Pharmaceutical properties	Physical properties	Disintegration time	≤3 min	Meeting the European Pharmacopoeia requirements on the disintegration times of ODTs; the USP requires that the disintegration time is limited to 30 s [9].
		Tensile strength	Sufficient to allow handling	There are no official values for the tensile strength of orodispersible dosage forms; the values mentioned previously have been used in practice.
		Friability	≤1%	Meeting the European Pharmacopoeia requirements as specified in monograph 2.9.7, Friability of uncoated tablets.
	Uniformity of dosage units		Unless otherwise specified, meeting the European Pharmacopoeia requirements as specified in the monographs on Uniformity of dosage units (2.9.40), Uniformity of content (2.9.6), Uniformity of mass (2.9.5) and Dissolution test for solid dosage units (2.9.3).	
	Uniformity of content			
	Uniformity of mass			
	Dissolution			
	Release profile		Immediate/prolonged release	According to the API and therapeutic indication of the drug product.
Acceptability		Size and appearance	Acceptable for the patient; size should not pose any risk to the patient	The acceptability of dosage forms is a subjective matter, influenced by the age, conditions and personal preferences of every patient.
		Taste and palatability	Acceptable for the patient	
		Ease of administration	Requiring minimal ex tempore preparation	The drug should be easy to handle and administer in accurate doses.
Safety of excipients			Safe for the target population and suitable for the dosage form	If details on excipient safety are unavailable, potential excipient-associated risks should be taken into consideration
Stability			2 years minimum	Special storage conditions (e.g., fridge storage) are less preferred; the packaging should contribute to maintaining the structural integrity and stability of the dosage form.

**Table 2:** Studies on the acceptability of ODx in paediatric populations.

Test Product	Age Range	API	Reference Product	Results	Reference
ODT	5–11 years	Ondansetron	Placebo	The ondansetron ODTs were effective in reducing postoperative nausea and vomiting, but less palatable compared to the placebo ODTs.	[85]
	≤12 years	Desloratadine	Desloratadine syrup	Most of patients’ caregivers were open to trying the ODT formulation and preferred it to the syrup due to the convenience and lack of messiness of the administration.	[86]
	≤5 years	Artemether-lumefantrine	Dihydroartemisinin-piperaquine tablets	Both formulations were similarly effective to antimalarial medication, but the artemether-lumefantrine ODTs showed an increased treatment adherence.	[87]
	2–59 months	Amoxicillin	Amoxicillin oral suspension	The ODT formulation was equivalent to the suspension in terms of acceptability and clinical outcome, but it determined a better treatment adherence.	[88]
ODMT	≤12 years	Enalapril	Not applicable	The formulation is compatible with several vehicles commonly used to increase acceptability in younger children and is acceptable to older children.	[89]
OL	5–15 years	Desmopressin	Desmopressin tablets	The OL was preferred over the tablet, significantly so in the patients aged 5 to 11 years; the efficacy and safety were similar to the tablet at lower doses.	[90]
ODF	newborns	Vitamin D	Vitamin D syrup	Even though it needed more frequent administration, the ODF was much more preferred by patients and parents alike.	[68]
	6 months–5 years	Placebo	Not applicable	The majority of children aged 3 and over, as well as the caregivers of the entire patient cohort, gave the ODF a positive rating in terms of acceptability.	[91]
	2 days–12 months	Placebo	Placebo glucose syrup	The ODF was deemed as non-inferior to the syrup in terms of acceptability and superior in the palatability and swallowability assessments.	[92]

ODT—orodispersible tablet; ODMT—orodispersible minitablet; OL—oral lyophilisate; ODF—orodispersible film.

**Table 4:** Orodispersible dosage forms authorised for paediatric use in the European Union.

API	Drug Class	Dosage Form	Commercial Name(s)	Minimal Usage Conditions
Amoxicillin/clavulanic acid	Antibiotic	ODT	Amoksiklav Quicktab	≥40 kg
Aripiprazole	Antipsychotic	ODT	Abilify, generics	≥13 years
Bilastine	Antihistaminic	ODT	Borenar, generics	≥6 years
Desloratadine	Antihistaminic	ODT OL	Aerius, Neoclarityn, generics	≥6 years
Desmopressin	Hormone analogue	ODT OL	Minirin Melt Minurin Flas	≥5 years
Dexamethasone	Steroidal anti-inflammatory	ODF	Isicort	≥3 months and 7 kg
Domperidone	Prokinetic	ODT	Domotil	≥12 years and 35 kg
Ebastine	Antihistaminic	OL ODT	Bactil Flas, Kestine, Kestinlyo generics	≥12 years
Ibuprofen	Non-steroidal anti-inflammatory	ODT	Nurofen	≥6 years
Lamotrigine	Antiepileptic	ODT	Lamictal	≥2 years
Loperamide	Antidiarrheic	ODT OL	Imodium, generics	≥2 years
Lorazepam	Anxiolytic	OL ODT	Tavor Expidet, Temesta Expidet, generics	≥6 years
Montelukast	Leukotriene receptor antagonist	ODG	Singulair	≥6 months
Morphine	Opioid analgesic	ODT	Carpos Akut	≥6 months
Ondansetron	Antiemetic	ODF	Setofilm	≥6 months
Oxycodone	Opioid analgesic	ODT	Oxygesic Dispersa	≥12 years
Paracetamol	Analgesic/antipyretic	ODT	Pinex Smelt	≥4 years
Prednisolone	Steroidal anti-inflammatory	ODT	Solupred	≥10 kg

ODT—orodispersible tablet; OL—oral lyophilisate; ODF—orodispersible film.

## Data Availability

Not applicable.
